# Postoperative effects on lower third molars of using mouthwashes with super-oxidized solution versus 0.2% chlorhexidine gel: A randomized double-blind trial

**DOI:** 10.4317/medoral.22622

**Published:** 2018-11-21

**Authors:** Ana Coello-Gómez, Selene Navarro-Suárez, José-María Diosdado-Cano, Francisco Azcárate-Velazquez, Patricia Bargiela-Pérez, María-Ángeles Serrera-Figallo, Daniel Torres-Lagares, Jose-Luis Gutiérrez-Pérez

**Affiliations:** 1 Master’s Degree in Oral Surgery, University of Seville; 2 Full Professor of Oral Surgery. Dental School. University of Seville; 3 Professor of Oral Surgery. Dental School. University of Seville. Head of Oral and Maxillofacial Surgery Service. “Virgen del Rocío” University Hospital

## Abstract

**Background:**

The main objective of the present study is to evaluate the effects and possible benefits with regard to the postoperative period of lower third molar extractions, comparing the intraalveolar application of a bioadhesive gel of 0.2% chlorhexidine (CHX) to the use of a mouthwash with a super-oxidized solution, (SOS) Dermacyn® Wound Care (Oculus Innovative Sciences lnc., California, USA).

**Material and Methods:**

A randomized double-blind study was carried out in 20 patients with a split-mouth design, with a total of 40 extractions of symmetrically impacted bilateral lower third molars. Patients were divided into two groups, a control group (C = 20) and an experimental group (D = 20). Any infectious complications, wound healing, plaque accumulation in the stitches, and presence of trismus and inflammation were evaluated using the distance between different facial points, at three, eight, and fifteen days after extraction. Pain, swelling, and amount of analgesics taken were evaluated using the VAS scale throughout the 15 days following extraction. Tolerance to treatment was evaluated using a verbal scale. Results were statistically compared using the Student’s t- and chi-squared tests.

**Results:**

No statistically significant differences were found between the two groups with regard to infectious complications, swelling, or wound healing. Use of analgesics and self-reported pain levels were slightly lower in the experimental group than in the control group during days 6 and 7 of the study (*p*<0.05). The global treatment tolerance was satisfactory and similar in both groups.

**Conclusions:**

Both CHX and SOS are effective at improving the postoperative period after extraction of lower third molars.

** Key words:**Lower third molar, postoperative, chlorhexidine gel, super-oxidized solution, wound healing.

## Introduction

Surgical extraction of the third molars is the most common procedure in oral surgery given the high incidence of dysodontiasis observed in these teeth (1). These procedures generally involve significant postoperative changes that are part of the surgical intervention and therefore are not considered complications (2). Nevertheless, many published studies seek to minimize these effects in order to obtain more rapid wound healing (3,4). 

Different studies state that the intensity of swelling and pain during the postoperative period of these interventions is one of the most important factors when evaluating patients’ satisfaction and perception of the surgical treatment received (5).

Clinical conditions linked to extraction of the lower third molars such as pain, swelling, and trismus have been observed (6). Complications such as alveolar osteitis, hematomas, and damage to nearby teeth or nerves have also been reported (7).

Various studies in the literature evaluate different therapeutic protocols to improve the postoperative period after extraction of the lower third molars by integrating or modifying different aspects: use of preoperative antibiotics, different flap designs, osteotomies using high- or low-speed instruments, healing by either first or second intention, use of ice during the postoperative period, and use of corticosteroids (both via systemic and oral administration) (8-11).

Wound healing is a physiological process through which the loss of integrity of the oral mucosa is recuperated and damaged tissues are repaired. Without proper wound healing, bacteria greatly increase the risk of fibrinolysis, swelling, and poor wound healing, which result from infections in the extraction site. The most effective method for decreasing the risk of infection locally is the use of topical or systemic agents that can help to eliminate oral bacteria (12).

Chlorhexidine is one of the pharmaceuticals most often used for this purpose. It is an antiseptic designed to reduce dental plaque and oral bacteria, and it has been shown to have an immediate bactericidal effect as well as long-term bacteriostatic action (13).

When administered *in vivo*, chlorhexidine has been shown to have antibacterial properties more immediate than those of other antiseptics used in the oral cavity. Numerous studies support its use, whether topically or by applying a gel, in the improvement of wound healing (2,4,14).

Dermacyn Wound Care (DWC, Oculus Innovative Sciences Netherlands BV, Sittard, Netherlands) is a super-oxidized solution (SOS) that has proved useful in preventing infection at the level of the bone and surrounding soft tissues, thereby facilitating and improving wound healing (15-18).

The active components of this solution are 99.98% super-oxidized water and less than 0.02% of different reactive species of chlorine and oxygen, including hypochlorous acid, sodium hypochlorite, sodium chloride, ozone, and chlorine dioxide. It should be notice that the total content of free chlorine available is low, ranging between 50–80 ppm. A SOS has bactericidal, virucidal, fungicidal, and sporicidal effects, and it is ready for use without further dilution or mixing. Moreover, it does not require manipulation or special disposal, and it has a shelf life of over 12 months (17,20).

This study aims to assess the effects of chlorhexidine gel (CHX) and the SOS, applying the former on the wound surface after extraction of the lower third molars, with the latter being administered via a mouthwash during the postoperative period. The assessed factors were pain levels, wound healing, and postoperative swelling.

## Material and Methods

**Figure 1:**
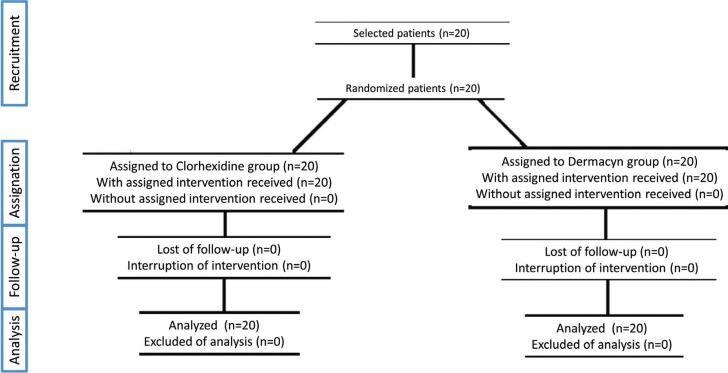
Flow chart of the study.

A randomized double-blind prospective study was carried out in 20 patients with a split-mouth design). Therefore, a total of 40 symmetrically impacted third molars were extracted. Patients were randomly divided into a control group (C = 20) and an experimental group (D = 20). The study recruitment period lasted from January to December 2016. Randomization was carried out for the first third molar of each patient using the website https://www.random.org/lists/. The second third molar of each patient was automatically assigned to the other group (Fig. 1).

Prior to extraction, the difficulty level was assessed using the Koerner scale, with the inclusion criteria for the present study necessitating a difficulty level from between 5-7, inclusively (21).

The exclusion criteria for the present study were: patients with any contraindications to surgery, immunocompromised patients, pregnant or breastfeeding women, women currently taking oral contraceptives, patients with allergies to chlorhexidine, lidocaine or paracetamol, patients who required the extraction of both third molars at the same time, presence of any type of bone pathology, and patients who had used antibiotics, anti-inflammatories, or analgesics within four days prior to surgery.

As initial variables, data such as gender, age, smoking habits, medical history, reason for extraction, and assessed difficulty level were recorded using the Koerner scale and a preoperative panoramic radiography (21).

Furthermore, the facial perimeter was measured during the preoperative period in order to give a better idea of the level of swelling observed after surgery. These measurements included the distance in millimeters from the inner corner (canthus) of the eye (OAT) to the outermost part of the ear (tragus) and from the labial commissure to the tragus (ComAT). The maximum mouth opening was also measured as the distance between the upper and lower incisal edges in millimeters.

During surgery, the total procedure time was recorded in minutes; any intraoperative complications were also recorded.

All of the extractions were performed by the same dentist in accordance with the surgical protocol used in this type of intervention (12). After finishing the extractions, a single dose of 0.2% chlorhexidine was administered to the socket before placing the suture (4-0 silk), or the SOS mouthwash was provided to the patient with enough for them to perform three rinses per day after brushing for one week. Patients were also provided a document with all postoperative instructions and the dates for follow-up appointments. A postoperative treatment of 400 g of ibuprofen every 8 hours for seven days was planned for both groups.

A different dentist, who was unaware of which treatment had been applied, performed the follow-up appointments of the patients. Infectious complications were measured on the third, eighth and fifteenth days, applying the criteria suggested in previous studies by our team (2,4), in addition to wound healing (wound consistency and appearance were categorized as good, acceptable, or bad (12), the presence of plaque on the surgical stitches (yes/no), and the maximum trismus, OAT and ComAT (following the same methodology used for the preoperative phase). Each patient recorded their levels of pain and swelling each day using a visual analog scale (VAS) from 0 to 10 throughout the 15-day follow-up period. Patients also made note of any rescue analgesics they needed to take. At the end of the study, the patients were asked to rate their tolerance to the treatment using a verbal scale.

For the sample calculation, a 95% level of confidence and 80% level of statistical power were used to identify a difference of 1 in the values of the VAS scale used to assess pain and/or swelling, assuming a variance of 1.25. In this way, the sample size was determined as 20 patients per group.

The data were included in a SPSS (IBM, USA) table for statistical analysis. The Kolmogorov-Smirnov test was used to measure data normality. The results of both groups were compared with the Student’s t-test for quantitative data and with the chi-squared test for all qualitative data.

The study was approved by the Ethics Committee of the University of Seville in Spain (CEI CODE: 2014PI7091), in accordance with the principles of the Helsinki Declaration. All patients received a document prior to surgery that described the study, signing their written informed consent to take part in the study and to undergo surgery.

## Results

The distribution of the initial variables was homogeneous in both groups, as all patients belonged to both the control and experimental groups. In the group that used CHX (C), 8 lower left third molars and 12 lower right third molars were extracted. In the SOS group (D), 12 lower left third molars and 8 lower right third molars were extracted. All patients complied with the set protocol. The mean age was 29.60 years ± 9.17. The studied sample consisted of 16 women and 4 men, with 5 smokers and 15 non-smokers.

Similarly, with regard to the intraoperative variables, the difficulty level of the lower third molars (Koerner scale) (21) selected was similar in both groups (5.35 ± 0.97 in the CHX group and 5.42 ± 0.86 using the Koerner scale of 0 to 10). The average procedure time of all surgeries was 17.45 minutes ± 4.94, and therefore no statistically significant differences were found between the two groups.

No statistically significant findings were observed during the follow-up appointment on the third day with regard to infectious complications (no infections were observed), wound healing during the postoperative period (all values were “good”), or plaque accumulation on the surgical stitches (no plaque was detected in any patient). The OAT, ComAT and incidence and severity of trismus were similar in both groups ( Table 1).

**Table 1:**
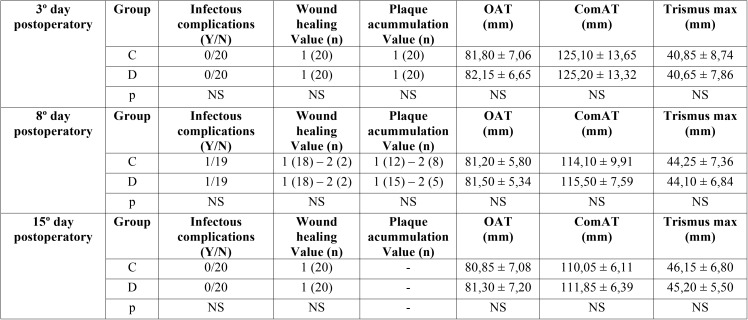
Data from the variables measured in the postoperative period: incidence of infectious complications; wound healing (possible values: 1, good; 2, regular; 3, bad); plaque accumulation on the surgical stitches (possible values: 1, yes; 2, no); OAT (distance in millimeters from the inner corner (canthus) of the eye to the outermost part of the ear) (mm); ComAT (distance in millimeters the labial commissure to the tragus ) (mm), and maximum trismus (mm) ; C Clorhexidine group ; D Dermacyn group) ; NS Non-significant difference; Y Yes ; N No.

On the eighth day an infectious complication with pus on the wound was observed when the suture was removed in both groups, with these wounds being subsequently treated as a result. The wound healing rate and presence of plaque on the sutures were similar in both groups; only two patients in each group showed “regular” wound healing. In the chlorhexidine group, two surgical stitches were observed to have plaque accumulation, with five patients in the Dermacyn®, or SOS, group exhibiting plaque accumulation. The OAT, ComAT and trismus values were similar between both groups, without finding any significant differences ( Table 1). The last follow-up appointment took place fifteen days after surgery, with no significant differences between the two groups reflected in the data ( Table 2).

**Table 2:**
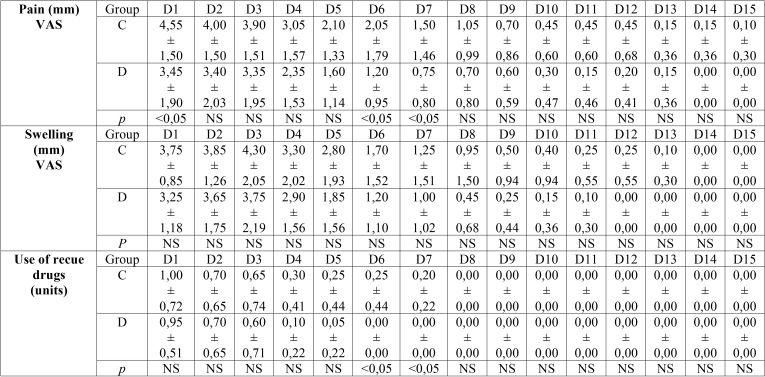
Data on pain and swelling (ranked from 0 to 10 using a VAS scale) and use of rescue drugs during the studied postoperative period. C Clorhexidine group; D Dermacyn group ; D1 to D15 Day 1 to day 15 of follorw-up; NS Non-significant difference; Y Yes ; N No ; VAS Visual analogic scale.

**Figure 2:**
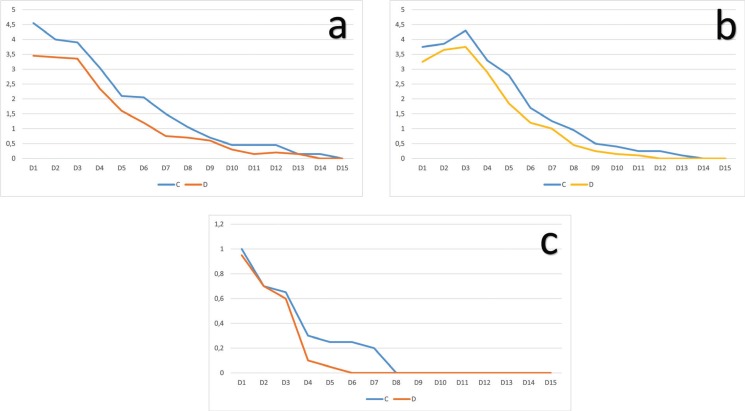
a) Pain during the 15-day postoperative period. C = Chlorhexidine group, D = Dermacyn® group; b) Swelling during the 15-days postoperative period. C = Chlorhexidine group, D = Dermacyn® group; c) Use of rescue medication during the 15-day postoperative period. C = Chlorhexidine group, D= Demarcyn® group.

With regard to postoperative pain during the 15 days’ follow-up period, the data showed that this was slightly lower in the experimental group (Dermacyn® or SOS) than in the control group (chlorhexidine) throughout the entire study. However, this difference was significant only on the first, sixth and seventh days ( Table 2, Fig. 2a). The swelling was also lower in the experimental group than in the control group during the entirety of the follow-up period, but no statistically significant differences were found between both groups ( Table 2, Fig. 2b). The data regarding rescue medication provided similar results; need for rescue medication was always lower in the experimental group when compared with the control group. However, significant differences between both groups were observed on the sixth and seventh day ( Table 2, Fig. 2c). The total treatment tolerance was satisfactory and similar in both groups.

## Discussion

When performing oral surgery, numerous types of oral antiseptics can be use as adjuvants. They are used to encourage tissue healing and obtain better postoperative results. Such antiseptics can be found in different compositions and different routes of administration. It is important to evaluate the different effects of administering these solutions either before or after the surgery, as well as with regard to the type of solution and route of administration.

Mouthwashes and gels are the most commonly used and requested by both clinicians and patients. They are used to decrease the microbial load in the oral cavity and can be used before or after oral or periodontal surgery (22).

Numerous scientific studies have shown that mouthwashes containing chlorhexidine digluconate (CHX) which have antibacterial properties and reduce bacteria in the oral epithelium (4,23,24).

Various microorganisms, such as Actinomyces naeslundii, Veillonella dispar, Prevotella nigrescens, and Streptococcus are highly susceptible to chlorhexidine (25).

Chlorhexidine in gel form allows it to be placed inside the socket, providing a more direct and long-term action when compared with chlorhexidine mouthwash, which is applied superficially. Other studies have assessed the intraalveolar application of CHX gel during the intraoperative period, which seems to decrease the incidence of alveolitis, or dry socket, after extraction of the lower third molars, even in patients with coagulation disorders (6,8,9,26).

No clinical trials have been published to date that are similar to the present study, in which a SOS was used in oral surgery. However, there are studies with experience using this solution as an adjuvant treatment to manage postoperative wounds of diabetic foot osteomyelitis in patients with a high risk of amputation. The majority of them conclude that the use of SOS as an adjuvant treatment in the postoperative is safe and can even eradicate the infection in the bone and surrounding soft tissues when combined with antibiotic therapy. However, authors agree that more studies are needed to confirm this (15,17,18).

Other studies have explored the use of SOS in avoiding postoperative infections, even in literature reviews conducted by Cochrane (20).

Therefore, since no other studies in the field of oral surgery have used a SOS, the present study carried out the first comparison of this compound with the gold standard used in these situations, chlorhexidine. Multiple studies can support this affirmation that chlorhexidine is the gold standard (27,28).

Osunde *et al.* carried out a study in 100 patients, applying a hot saline solution in the control group and 0.12% chlorhexidine mouthwash in the experimental group. The authors did not find statistically significant differences between the use of hot saline solution and 0.12% chlorhexidine mouthwash with regard to the development of alveolar osteitis in patients who had undergone third lower molar extraction (27).

Kaur *et al.* performed a prospective double-blind study with a total of 300 impacted molars in 150 patients. In the study group, the combination of metronidazole with chlorhexidine gel was introduced. In the study group, the incidence of alveolitis was significantly reduced, from 22.6% to 6.6% (*p* ≤ 0.001); therefore, they concluded that the intraalveolar use of this gel decreases the incidence of adverse reactions and complications related to the extraction of lower third molars (28).

In the present study, the study sample is adequate. Moreover, the study design makes the distribution of all variables exact in both groups, as all patients are classified as both control and experimental at the same time. In other words, the distribution of gender, age, allergies, and habits are similar in both groups. Likewise, it has been confirmed that the difficulty level of the third molars selected (Koerner scale) is similar, and the same individual belongs to the control and study group, which makes for a homogeneous sample.

The data collected in this study confirm that the treatment with a SOS shows at least the same results as the CHX group with regard to infectious complications, swelling, pain, healing, and need for rescue medication. Our graphics show that the group treated with a SSO is ahead of the CHX group. While it is true that such differences are small and over a short period of time, and therefore of limited clinical significance, even with a sample of 40 extractions, differences in pain and analgesic consumption were observed on days 6 and 7, matching the end of the postoperative period. It could be concluded that the recovery is slightly better and quicker with a SSO than with CHX.

Regarding mouth opening and pain, no significant differences were observed. None of the side effects reported by other similar studies were observed, and no complications arose (6,12,29).

The antiseptic activity of the compound, as well as its low toxicity, may have influenced the results of the study, as both characteristics are below other antiseptics such as Betadine, which has been assessed in other recent published studies (30).

Given the minimal clinical difference in the use of these antiseptics, it would be interesting to increase the number of studies and sample size to evaluate other secondary variables, such as the occurrence of specific side effects, as well as to include the economic cost evaluation of the product in order to provide a broader assessment of both pharmacological presentations.

In conclusion, it can be said that the data of the present study support that the use of 0.2% chlorhexidine gel and the super-oxidized solution Dermacyn® is effective and efficient in improving postoperative healing after extraction of the lower third molars.
